# Identification and Characterization of an Anti-Fibrotic Benzopyran Compound Isolated from Mangrove-Derived *Streptomyces xiamenensis*

**DOI:** 10.3390/md10030639

**Published:** 2012-03-15

**Authors:** Min-Juan Xu, Xiao-Jin Liu, Yi-Lei Zhao, Dong Liu, Zhen-Hao Xu, Xiao-Meng Lang, Ping Ao, Wen-Han Lin, Song-Lin Yang, Zhi-Gang Zhang, Jun Xu

**Affiliations:** 1 Shanghai Center for Systems Biomedicine, Shanghai Jiao Tong University, Key Laboratory of Systems Biomedicine, Shanghai 200240, China; Email: minjuanxu@sjtu.edu.cn (M.-J.X.); aoping@sjtu.edu.cn (P.A.); 2 Department of Plastic Surgery, Shanghai Jiaotong University Affiliated Sixth People’s Hospital, Shanghai 200233, China; Email: xjliu00058065@gmail.com (X.-J.L.); yangsonglin1961@gmail.com (S.-L.Y.); 3 State Key Laboratory of Oncogenes and Related Genes, Shanghai Cancer Institute, Ren Ji Hospital, Shanghai Jiao Tong University School of Medicine, Shanghai 200240, China; 4 State Key Laboratory of Microbial Metabolism and School of Life Science & Biotechnology, Shanghai JiaoTong University, Shanghai 200240, China; Email: yileizhao@sjtu.edu.cn (Y.-L.Z.); bao2lu@gmail.com (Z.-H.X.); 5 State Key Laboratory of Natural and Biomimetic Drugs, Peking University, Beijing 100191, China; Email: ldddddd@yahoo.cn (D.L.); lxmdyt112@sina.com (X.-M.L.); whlin@bjmu.edu.cn (W.-H.L.)

**Keywords:** *Streptomyces xiamenensis*, mangrove, benzopyran, fibroblast, anti-fibrosis, anti-contractile capacity

## Abstract

An anti-fibrotic compound produced by *Streptomycesn xiamenensis*, found in mangrove sediments, was investigated for possible therapeutic effects against fibrosis*.* The compound, *N*-[[3,4-dihydro-3*S*-hydroxy-2*S*-methyl-2-(4′*R*-methyl-3′*S*-pentenyl)-*2H*-1-benzopyran-6-yl]carbonyl]-threonine (**1**), was isolated from crude extracts and its structure, including the absolute configuration was determined by extensive spectroscopic data analyses, Mosher’s method, Marfey’s reagent and quantum mechanical calculations. In terms of biological effects, this compound inhibits the proliferation of human lung fibroblasts (WI26), blocks adhesion of human acute monocytic leukemia cells (THP-1) to a monolayer of WI26 cells, and reduces the contractile capacity of WI26 cells in three-dimensional free-floating collagen gels. Altogether, these data indicate that we have identified a bioactive alkaloid (**1**) with multiple inhibitory biological effects on lung excessive fibrotic characteristics, that are likely involved in fibrosis, suggesting that this molecule might indeed have therapeutic potential against fibrosis.

## 1. Introduction

Fibrotic diseases such as pulmonary fibrosis, cirrhosis, systemic sclerosis, interstitial nephritis and cardiovascular fibrosis are a threat to public health [1]. Constant high exposure to respirable particles is known to directly cause chronic inflammation-associated pulmonary diseases, including lung fibrosis and lung cancer [2]. These observations have resulted in increased research into mechanisms associated with fibrosis, the excessive fibrous connective tissue that underlies many chronic inflammatory pulmonary pathologies [1]. Indeed, studies have suggested that about 45% of deaths in developed countries are associated with fibrotic diseases of various organs [1]. 

Fibroblasts, one of the most abundant cells in the interstitial tissues, are known to play an important role in inflammation and fibrogenesis [1,3]. Interactions between fibroblasts and the local microenvironment can induce the formation of self-perpetuating circuits of inflammation [4]. These circuits can maintain the prolonged and elevated excellular matrix (ECM) secreting phenotype of fibroblasts, which is the defining characteristic of fibrotic diseases. Therefore, the search for leading compounds, particularly those obtained from a natural resource, that target fibroblasts represents an emerging pharmacological and therapeutic focus against fibrotic diseases.

Mangrove and its derived actinobacteria strains, are well-known for producing novel secondary metabolites and possessing highly effective bioactive compounds, possibly due to biochemical adaptation to the special intertidal ecosystem [5,6,7,8,9]. For example, in studies of anti-inflammatory compounds, 7-deacetylgedunin from *Xylocarpus moluccensis* was found to inhibit nitric oxide production in activated macrophages [10]. In another study, diterpenoids from *Excoecaria agallocha* L. were observed to suppress expression of NF-κB and AP-1 (activating protein-1) targeted genes including TNF-α and IL-6 in mouse macrophages [11]. Surfactin isomers from the mangrove bacterium *Bacillus* sp. were also tested on the previous two models [12]. Finally, a mixture of *Acanthus ilicifolius* leaf extract was also shown to exhibit inhibitory activity in rat paw edema [3]. The immense and untapped microbial biodiversity in the mangrove is a promising resource for marine-derived leading therapeutic compounds. 

**Figure 1:**
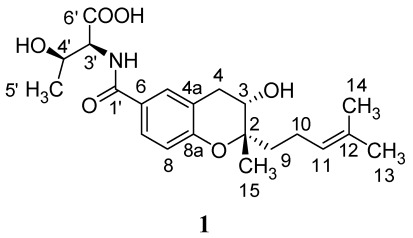
Structure of compound **1** isolated from *Streptomyces xiamenensis*.

*Streptomyces xiamenensis *strain 318 was identified as a novel species of actinobacteria from mangrove sediments [13]. During our preliminary screening for anti-fibrotic compounds, a crude extract of *S. xiamenensis *was tested in an adhesion-bioassay of fibroblasts on monocytes, and showed inhibitory activity. Further studies were then carried out to identify and characterize the active ingredient in this extract. In our work, an alkaloid, *N*-[[3,4-dihydro-3*S*-hydroxy-2*S*-methyl-2-(4′*R*-methyl-3′*S*-pentenyl)-*2H*-1-benzopyran-6-yl]carbonyl]-threonine (**1**), was isolated from crude extracts of *S. xiamenensis* (Figure 1.) and demonstrated to have considerable bioactivity. Compound **1** was previously isolated from *Streptomyces* sp. Mer-88, and shown to inhibit binding between LFA-1 and ICAM-1, and suggested to have important anti-inflammatory properties [14]. The absolute configurations of compound** 1**, including four stereogenic centers, were determined by extensive spectroscopic data analyses, Mosher’s method, Marfey’s reagent and quantum mechanical (QM) calculations. Compound **1** was found to exhibit inhibitory effects on cell proliferation of human lung fibroblasts (WI26), the contractile capacity of WI26 cells in a three-dimensional free-floating collagen gels, and the adhesion of human acute monocytic leukemia cells (THP-1) on a WI26 monolayer. As these properties are thought to contribute to the development of fibrosis, these data suggest that the compound described herein might indeed prove to be of considerable therapeutic benefit against fibrotic pathologies.

## 2. Results and Discussion

### 2.1. Isolation and Structural Elucidation of Compound 1

Crude extracts from *S. xiamenensis *were assayed for their inhibitory effect on the adhesion of THP-1 cells on a monolayer of WI26 cells. After incubating the crude extract (50 μg/mL) for 3 h, the adhesion of the THP-1 cells was about 15% lower than that with the solvent control (0.01% DMSO). Thus, the extract showed inhibitory adhesion activity and *S. xiamenensis* was considered as a promising bioactive resource for further chemical analyses. Guided by this bioactivity, a series of alkaloids were detected, using TLC and UPLC-MS, as the main secondary metabolites from the mixture extracted in a solution of ethyl acetate: methanol: formic acid = 80:15:5. The presence of pseudo-molecular ions [M + H]^+^ at *m*/*z *392.2069 and [M − H]^−^ at *m*/*z *390.1886 in positive and negative ion modes, respectively, in the mass spectra, led to the purification of the main component, compound **1**. 

The molecular formula of **1** is C_21_H_29_NO_6_ based on HRESIMS, which is in accordance with the previous literature [14]. In the ^1^H NMR spectrum of **1**, three aromatic protons with signals at δ_H_ 7.63 (1H, d, *J *= 8.4 Hz), 6.81 (1H, d, *J *= 8.4 Hz), 7.76 (1H, s), and 7.78, (1H, d, *J *= 7.8 Hz), as well as a high-field double-bond proton at δ_H_ 5.12 (1H, dd, *J *= 7.1, 1.3 Hz) were detected (Table 1). Carbon signals in the ^13^C NMR spectrum consisted of six aromatic carbons, plus two olefinic carbons, two carbonyl groups, two hydroxylated carbon signals at δ_C_ 79.8, 66.3, and a methylene at δ_C_ 31.2 (Table 1), which is in accordance with the structure of a benzopyran moeity, attached to two side chains, threonine and isoprenylmethyl group. The HMBC correlations (Table 1) further confirmed the planar structure of **1**, named *N*-[[3,4-dihydro-3-hydroxy-2-methyl-2-(4′-methyl-3′-pentenyl)-2H-1-benzopyran-6-yl]carbonyl]-threonine [14]. 

**Table 1:** ^1^H and ^13^C NMR spectroscopic data of compound** 1** and Δδ^RS^ values of key H-atoms in (*R*)- and (*S*)-MPA diesters ^a^.

Position	1			*R*/*S*-MPA-1		
	δ_H_ (*J* in Hz)	δ_C_, type	HMBC	δ^R^	δ^S^	Δδ^RS^
1	--	--				
2	--	79.8, C				
3	3.77, t ^b^	66.3, CH	4a, 2, 9, 15			
4	2.71, dd (17.3, 7.4)	31.2, CH_2_	8a, 5, 4a, 2, 3	2.54	2.82	−0.28
	2.98, dd (17.3, 5.2)			3.09	3.23	−0.14
4a	--	120.6, C				
5	7.67, s	129.8, CH	7, 8a, 4, 1′			
6	--	126.0, C				
7	7.63, d (8.4)	127.2, CH	5, 8a, 1′			
8	6.81, d (8.4)	116.7, CH	4a, 8a, 6			
8a	--	156.1, C				
9	1.60, m	38.0, CH_2_	11, 12, 2,3	1.55	1.29	+0.26
				1.47	1.21	+0.26
10	2.10, m	21.6, CH_2_	11, 12	2.05	1.95	+0.10
11	5.12, dd (7.1, 1.3)	124.8, CH	13, 10, 14, 9	5.01	4.89	+0.12
12	--	131.3, C				
13	1.57, s	17.9, CH_3_	11	1.51	1.47	+0.04
14	1.65, s	25.9, CH_3_	11	1.60	1.58	+0.02
15	1.18, s	18.7, CH_3_	2, 3, 9	1.27	0.97	+0.30
1′	--	166.6, C				
2′	7.78, d (7.8)	--	1′	8.29	8.40	−0.11
3′	4.38, brd	58.9, CH	4′, 6′	4.81	4.84	−0.03
4′	4.18, brs	67.1, CH				
5′	1.12, d (6.0)	20.9, CH_3_		1.27	1.10	+0.17
6′	--	172.8, C				

^a^ Measured in DMSO-*d*_6_, Chemical shifts (δ) in ppm; ^b^ Measured in [D_4_]-methanol, δ_H_ 3.88, dd (7.4, 5.2).

### 2.2. Absolute Configurations of Compound 1

**Figure 2:**
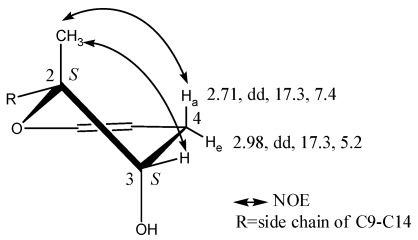
Key NOE correlations of the dihydropyran moiety in compound** 1**.

The absolute configurations of **1**, with four stereogenic centers, were identified by Mosher’s method, Marfey’s reagent, NOESY experiment, and QM calculations. Compound **1** was treated with (*R*)- and (*S*)-α-methoxy-α-phenylacetic acid (MPA) to form (*R*)- and (*S*)-MPA diesters. Diagnostic Δδ^RS^ sigals of the (*R*)- and (*S*)-MPA diesters resulted in negative Δδ^RS^ values for 4-H_2_ (Table 1), which were in contrast to the positive Δδ^RS^ values for 9-H_2_, 10-H_2_, 11-H, 13-H_3_, 14-H_3_ and 15-H_3_, which are all related to the dihydropyran moiety. These data are in accordance with a 3*S* configuration of** 1**. The absolute configuration of the neighboring position 2 was then determined by elucidating the relative configuration related to 3*S*. The NOE correlation between 2-H_3_ and 4-H_a_ demonstrated that 2-H_3_ was in an axial orientation. The other NOE interaction between 2-H_3_ and 3-H demonstrated that 3-H was equatorially oriented and 3-OH was in a *trans *configuration to 2-H_3_ (Figure 2). The relative configuration of 2-H_3_, 3-H and 4-H_a/e_ were further confirmed from the values of the coupling constants, which were 4-H_a_ (2.71, dd, 17.3, 7.4) and 4-H_e_ (2.98, dd, 17.3, 5.2). 

QM calculations on a model molecule of 2,2-dimethyl-3,4-dihydro-2*H*-pyran-3-ol indicate that, compared to that at the equatorial position, the 3-OH group prefers the axial position by about 1.2 kcal/mol both in the gas phase and in DMSO solvent due to the gauche effect [15]. In the gauche conformation, the H-H distances of 2-H_3_ are as close as 2.55 Å to 3-H, and 2.30 Å to 4-H_a_. The results are consistent with the key NOE correlations (Figure 2). Based on the absolute configuration of C-3 and the relative configuration of C-2, we therefore identified the absolute configuration of position 2 to be 2*S*.

As far as the other two stereocenters in the threonine moiety, diagnostic Δδ^RS^ sigals of the (*R*)- and (*S*)-MPA diesters resulted in negative Δδ^RS^ values for 3'-H and 2'-NH (Table 1), which were in contrast to the positive Δδ^RS^ values for 5'-H. The configuration of the position 4' was thus assigned to be *R*. In order to determine the configuration at the position 3', **1 **was hydrolyzed in hydrochloric acid and then derivatized with 1-fluoro-2,4-dinitrophenyl-5-L-alanine (FDAA, Marfey’s reagent) [16]. Based on the results of hydrolyzation of **1** and derivatization with Marfey’s reagent afterwards, the threonine moiety was assigned as the naturally occurring structure of L-threonine [(2*S*,3*R*)-2-amino-3-hydroxy butanoic acid]. The absolute configuration of **1** was therefore determined to be *N*-[[3,4-dihydro-3*S*-hydroxy-2*S*-methyl-2-(4′*R*-methyl-3′*S*-pentenyl)-*2H*-1-benzopyran-6-yl]carbonyl]-threonine (**1**).

### 2.3. Inhibition of Proliferation on Human Diploid Lung Fibroblast (WI26) Cells by Compound 1.

**Figure 3:**
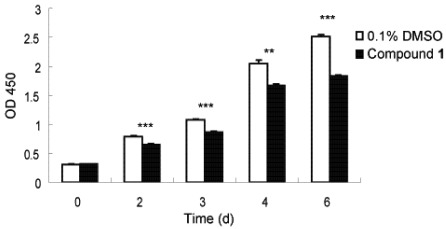
Inhibitory effect of compound **1** on WI26 cells proliferation. The WI26 cells were exposed to 30 µg/mL of **1** at day 0, 2, 3, 4 and 6. Surviving fraction was determined by Cell Counting Kit-8 assay. As illustrated, proliferation of WI26 cells was significantly inhibited by **1** in a time-dependent manner. Data are given as the mean of triplicate values ± SD of three independent experiments. Significant differences from the value of 0.1% DMSO solvent control were marked **p < 0.01, ***p < 0.001.

Fibrosis is characterized by the overgrowth, hardening, and/or scarring of various tissues and is attributed to an excess deposition of ECM components including collagen [1]. To combat this, an ability to slow down rapid fibrotic response during typical wound repair and tissue regeneration processes would seem to be a reasonable intervention, with the goal to avoid an accumulation of non-functional fibrotic tissue and thereby enable transformation to more regenerative conditions [3]. We thus investigated the anti-proliferative activity of **1** to this end. Lung fibroblast WI26 cells were exposed to **1**, at a concentration of 30 µg/mL, for 0, 2, 3, 4 and 6 days, and the optical density (OD) λ = 450 nm was monitored as a function of time. As illustrated in Figure 3, compound **1** significantly inhibited the proliferation of WI26 cells. The extent of this inhibition increased with time (Figure 3), from a decrease of 7% at day 2 to 27% at day 6, compared with solvent control. Compound **1** therefore indeed exhibits anti-proliferative effects on lung fibroblast cells.

### 2.4. Effect of Compound 1 on the Adhesion of THP-1 Cells to a Monolayer of WI26 Cells

**Figure 4:**
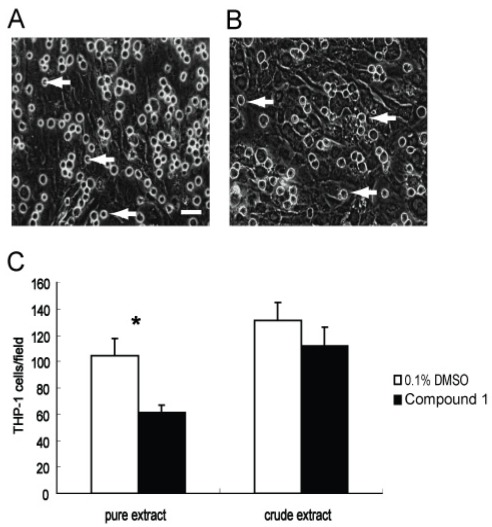
Blocking adhesion of THP-1 cells onto a monolayer of WI26 cells by compound **1**. Representative pictures of the adhering cells taken with 400 fold magnification. Equal numbers of THP-1 cells with **1** (30 μg/mL) or 0.1% DMSO were added to a monolayer of WI26 cells in triplicate wells of 24-well plates, respectively. Adhering THP-1 cells after 3-h-incubation were counted in four random visual fields of each well. (**A**) The attachment of THP-1 cells (marked by arrows) onto confluent WI26 cells, treated with 0.1% DMSO; (**B**) The attachment of THP-1 cells onto confluent WI26 cells, treated with** 1** (30 μg/mL). It is clear from these images that adhesion of THP-1 cells was significantly blocked by **1**. Bar = 25 μm. Each point represents the mean ± SD of three independent experiments. Significant difference from the value of 0.1% DMSO solvent control was marked, *p < 0.05.

Monocytes and macrophages have been shown to play pivotal roles in inflammation and fibrosis by modulating the secretion of cytokines as well as recruiting and activating fibroblasts and other inflammatory cells [17]. Direct contact between monocytes/macrophages and fibroblasts is important for the initiation, perpetuation, and resolution of fibrosis [18]. We therefore also evaluated the anti-adhesion bioactivity of **1**. WI26 cells were first grown as a cell monolayer on a rigid tissue culture support, and then monocytic THP-1 cells were added to this monolayer. The number of adhered cells was then measured by optical microscopy after a 3 h incubation with either **1** (30 μg/mL) or 0.1% DMSO. As shown in Figure 4, there were obvious differences between the experiments with **1** present compared with those of the solvent control (Figure 4). Namely, the adhesion of THP-1 was about 41% lower in the presence of **1** than that in the solvent control. Thus, compound** 1** exhibits significant anti-adhesion bioactivity for monocytes on fibroblasts.

### 2.5. Effects of Compound 1 on Contraction of WI26 Cells in 3D Collagen Lattices

**Figure 5:**
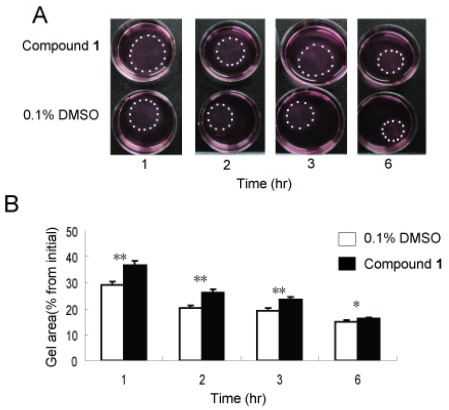
Compound **1** inhibited contractile ability of WI26 cells in 3D collagen lattices. Equal numbers of WI26 cells were seeded in triplicate gels of collagen I with compound **1** (30 μg/mL) or 0.1% DMSO. The contraction of collagen gel was monitored by photographing the gels at appointed time intervals. (**A**) As illustrated, contraction of collagen gel was attenuated by compound** 1** compared to 0.1% DMSO at different time points. The edge of gel was marked with dots. (**B**) The area of the gel was measured and plotted as a percentage of the original area at the onset of the experiments. Each point represents the mean ± SD of three independent experiments. Significant differences from the value of 0.1% DMSO solvent control were marked, * *p* < 0.05, ** *p* < 0.01.

It is widely known that mechanical force plays a contributing role in inflammation and fibrotic processes [19,20]. Not surprisingly, the contractible force generated by fibroblasts is a focus of much research [21]. Activated fibroblasts express many stress fibers which can facilitate wound repair [3] and fibrogenesis [22], while at the same time mechanical stress can promote inflammation and fibrosis by activating fibroblasts and inflammation cells [19,23]. In order to evaluate the anti-contractility activity of **1**, three-dimensional collagen lattices were used as a richer, perhaps more physiologically relevant physical environment, that can enable direct investigation of the interactive process of fibroblast-collagen matrix remodeling [24]. WI26 cells were seeded in free-floating collagen gel lattices, followed by the application of **1 **or solvent control. As shown in Figure 5A, the presence of compound **1** significantly delayed contraction of the collagen gel. After 1, 2, 3 and 6 h, the contraction was markedly decreased compared to solvent control by 21%, 22%, 18% and 8%, respectively (Figure 5B). 

### 2.6. Discussion

Today, fibrotic diseases are becoming more threathening than ever, due to enviromental pollution. It has been reported that high levels of air pollutant paticles can induce chronic fibrotic responses in airways [25]. Identification of bioactive molecules that affect fibrosis is thus becoming a promising theraputic area and also an urgent task. For example, the synthetic drug TAS-301, a constrictive remodeling regulator, was shown to have high inhibitory potency on coronary artery stenosis, mainly due to inhibition of adventitial fibroblast proliferation and the contractile ability of myofibroblasts [26]. During excessive fibrogenesis, it is well known that self-perpetuating circuits of inflammation and ECM accumulation, and constriction by inflammation and mechanical force, directly influence the development of fibrotic diseases [4]. Hence, the development of leading compounds aimed at these circuits may target the effects on these diseases, while avoiding severely disturbing physiological processes induced by steroidal anti-inflammatory drugs.

Fibroblasts play pivotal roles in establishing and maintaining these circuits and are in fact the main ECM generating cells in fibrotic diseases [27]. During these diseases, resident fibroblasts proliferate and are activated as a result of stimulation by injury or inflammation. The activated fibroblasts contribute to ECM production and inflammatory modulation [28]. Radiotherapy and chemotherapeutics are useful for treating hypertrophic scar and keloid development by inducing apoptosis of fibroblasts [29]. Compound **1** showed inhibitory effects on fibroblast proliferation, and thus may contribute to the alleviation of excessive fibrotic accumulation.

Activated fibroblasts may also convert into myofibroblasts, which express many more stress fibers and generate much more mechanical tension [1]. The mechanical force generated by endogenous fibroblasts has been found to play important roles in the activation and secretion of inflammatory cells [19,23,30]. It has been demonstrated that reduction of mechanical stress can alleviate inflammation and fibrosis *in vivo* [12]. Therefore, mechanical force and local inflammation together comprise one of the most important self-perpetuating inflammatory circuits that, underlies many fibrotic diseases. Compound **1 **can reduce the contractile capability of fibroblasts, thus indicating a potential to inhibit this key aspect of fibrogenesis.

The interaction of monocytes and fibroblasts is known to play an important role in inflammation [17,31]. Direct contact between monocytes and fibroblasts can faciliate the activation of both cells [32]. Compound **1** blocked the adhesion of fibroblasts and monocytes, which may ameliorate excessive inflammatory processes in fibrotic diseases. In previous investigation,** 1 **was reported as an antagonist of the LFA-1/ICAM-1 interaction [14]. Since LFA-1/ICAM-1 can mediate direct contact of inflammatory cells and fibroblasts [33], the LFA-1/ICAM-1 binding inhibitory effect may explain the results of the adhesion experiment.

In conclusion, **1** has multiple biological effects on lung fibroblast properties, including proliferation, contractile capacity and interaction with monocytes, which are likely to be involved in the establishment of positive feedback circuits that lead to excessive fibrosis. 

Although pathological fibrosis of different organs is common in many diseases [1], there are presently very few therapeutic options [34]. Anti-inflammatory drugs such as corticosteroids can prevent pathological fibrosis [29,35], but long-term use and high dosage are unsuitable owing to their extensive adverse effects [36,37]. Superficial compression is useful for preventing the development of skin hypertrophic scars [29], but this procedure cannot be used in visceral organs. The “vicious cycle” of fibroproliferation is one of the most important characteristics of pathological fibrosis [19,23]. Hence, small molecules that target the positive feedback circuits may prove tremendously effective in exhibiting antifibrotic activities without severe side effects. The results presented herein illustrate that compound **1** may serve as a useful antifibrotic leading compound for treating pulmonary fibrosis and other pathological fibrotic diseases.

## 3. Experimental Section

### 3.1. General

^1^H and ^13^C NMR spectra were recorded with Bruker DRX-500 and Avance III-600 NMR spectrometers with the solvent as an internal standard (DMSO-*d*_6_ δ = 2.51 and 40.0 ppm, respectively). Chemical shifts (δ) expressed in parts per million (ppm) and coupling constants (*J*) are reported in Hertz (Hz). LC-HRMS was performed on a Waters ACQUITY UPLC system equipped with a binary solvent delivery manager and a sample manager, coupled with a Waters Micromass Q-TOF Premier Mass Spectrometer equipped with an electrospray interface (Waters Corporation, Milford, MA). Column chromatography was performed with silica gel (200–300 mesh, Qingdao Marine Chemical, Inc., Qingdao, China), silica gel H (10–40 μm, Qingdao), Sephadex LH-20 (40–70 μm, Amersham Pharmacia Biotech AB, Uppsala, Sweden) and Lichroprep reversed-phase RP-18 silica gel (40–63 μm, Merck, Darmstadt, Germany). The chemical reagents used for chromatography were purchased from Shanghai Chemical Works Co. Ltd. (Shanghai, China). Optical rotations were recorded on a JASCO P-2000 polarimeter. CD spectra were taken on a J-815 spectropolarimeter (JASCO, Gross-Umstadt, Germany) at room temperature. An Agilent 1200 series system was used for the analytical scale method. Analytical HPLC was carried out on an Welch XB-C18 column (4.6 × 150 mm, 5 μm, Welch Materials, Inc., USA), flow 1 mL/min. Organic solvents for HPLC were analytical grade and were purchased from Shanghai ANPEL Scientific Instrument Co. Ltd. (Shanghai, China).

### 3.2. Strain Cultivation

*Streptomyces xiamenensis *strain 318 was isolated from a mangrove sediment sample collected in the national mangrove reserve in Fujian province of China [13]. Spores of strain 318 were inoculated on GYM (Glucose 4 g, Yeast extract 4 g, Malt extract 10 g, CaCO_3_ 2 g, Agar 12 g, Distilled water 1 L, adjusted to pH 7.2 with KOH before adding agar.) plates were then incubated at 28 ± 1 °C for 12 days before harvesting. 

### 3.3. Extraction and Isolation

The 30 L plate culture of *S. xiamenisis* was extracted with a solvent mixture of ethyl acetate/methanol/acetic acid (80:15:5, v:v:v) at room temperature overnight. The supernatant was filtered and the residue was then extracted twice as described above. The total supernatants were combined and concentrated under vacuum at 37 °C in order to remove the organic part to afford a crude extract (12 g). Half of the crude extract was subjected to column chromatography on silica gel, eluted by CH_2_Cl_2_:MeOH (gradient from methanol, 100:1, 50:1, 20:1, 10:1, 5:1 to MeOH). The fraction [eluted with CH_2_Cl_2_:MeOH (10:1, v:v)] was collected and purified by repeated column chromatography on Sephadex LH-20, which was washed by MeOH. The fractions were collected and guided by HPLC fingerprints to find peaks containing the target UV profile (λ_max_ 206, 260 nm). Finally, the combination was separated by ODS column (eluted with MeOH:H_2_O = 75:25) to obtain compound **1** (15 mg). 

*N*-[[3,4-dihydro-3*S*-hydroxy-2*S*-methyl-2-(4′*R*-methyl-3*S*-pentenyl)-*2H*-1-benzopyran-6-yl]carbonyl]-threonine (**1**): Yellow amorphous powder (MeOH); [α]^22^_D_ +39.5° (*c *0.044, MeOH); UV λ_max_ (MeOH) 206, 260 nm; CD (MeOH) Δε_201_ +0.15, Δε_202_ +0.55, Δε_205_ +0.3, Δε_207.5_ +0.5, Δε_229_ −0.05, Δε_259_ +0.28; ^1^H and ^13^C NMR data, see Table 1; HRESIMS *m*/*z *392.2069 [M + H]^+^, (calcd for C_21_H_30_NO_6_, *m*/*z *392.2073), 390.1886 [M − H]^−^, (calcd for C_21_H_29_NO_6_, *m*/*z *390.1917).

### 3.4. (*R*)- and (*S*)-MPA Esterification

(*R*)-MPA (4.0 mg), dicyclohexylcarbodiimide (DCC), and catalytic *N*,*N*-dimethylpyridin-4-amine (DMAP) were added to a CHCl_3_ solution of compound **1**. The mixture was stirred for 2 h to yield (*R*)-MPA ester of **1**. The (*S*)-MPA ester (1.1 mg) was obtained by the same protocol as for (*R*)-MPA ester (1.2 mg). 

### 3.5. Hydrolysis of Compound 1, Derivatization with Marfey’s Reagent

Compound **1** (1.0 mg) was hydrolyzed in 1:1 hydrochloric acid-acetic acid at 100 °C for 18 h, 500 μL H_2_O was added and the mixture was lyophilized overnight [16]. Then 200 μL of 1 M NaHCO_3_ and 400 μL of 38.7 μM FDAA (Sigma-Aldrich, St. Louise, MO, USA) in acetone were added to the mixture for derivatization. The solution was vortexed and incubated at 40 °C for 60 min. Reactions were quenched by addition of 100 μL of 2M HCl. Samples were diluted 1:2 with HPLC initial mobile phase. The derivatization of L-Threonine (Sangon, Shanghai, China) and D-Threonine (Sangon, Shanghai, China) were prepared in the same way. Then the samples were analyzed by HPLC with UV detection at 340 nm and further confirmed by UPLC-MS.

### 3.6. Cell Culture

The WI26-SV40 transformed human lung fibroblast [American Type Culture Collection (ATCC) CCL 95.1] cells were maintained in Dulbecco's Modified Eagle Medium (DMEM, Gibco, Grand Island, NY, USA) supplemented with 10% fetal bovine serum (FBS, GIBCO-BRL, Gaithersburg, MD, USA), 2 mM L-glutamine, 100 U/mL penicillin and 100 μg/mL streptomycin (Solarbio, Beijing, China). THP-1 (human acute monocytic leukemia cell line) cells were maintained in RPMI 1640 meted with 10% fetal bovine serum (FBS, GIBCO-BRL, Gaithersburg, MD, USA), 2 mM L-glutamine, 100 U/mL penicillin and 100 μg/mL streptomycin (Solarbio, Beijing, China). THP-1 (human acute monocytic leukemia cell line) cells were maintained in RPMI 1640 medium (Gibco, Grand Island, NY, USA), supplemented with 10% FBS, 2 mM L-glutamine, 100 U/mL penicillin, 100 μg/mL streptomycin, and 0.5 mM/L β-mercaptoethanol (Gibco, Grand Island, NY, USA). Both kinds of cells were incubated at 37 °C humidified atmosphere of 5% CO_2_ in air.

### 3.7. Cell Proliferation Assay

The effect of compound **1 **on cell proliferation was determined using a standard Cell Counting Kit-8 (CCK-8, Dojindo, Kumamoto, Japan) assay according to the manufacturer’s instruction. WI26 cells at 70%–80% confluent were trysinized by 0.25% trypsin-0.02% EDTA solution, centrifuged and re-suspended in DMEM supplemented with 10% FBS and antibiotics and seeded in 96-well plates (100 μL/well) at an initial density of 2.5 × 10^4^ cells/mL. The medium was replaced 24 h later by fresh DMEM with 10% FBS and antibiotics containing 30 μg/mL compound **1** or 0.1% DMSO (AppliChem, Darmstadt, Germany), then the medium was refreshed and the cells viability was measured by using CCK-8 solution at day 0, 2, 3, 4 and 6, respectively. Proliferation measurement was applied by adding 10 μL CCK-8 solutions to each well and incubating at 37 °C for 1 h. The OD values of each well were measured at the primary wavelength λ = 450 nm by using a Microplate Spectrophotometer (PowerWaveXS, BioTek, Seattle, WA, USA). Data are shown as means ± standard deviation (SD) of three independent experiments, each performed in triplicate.

### 3.8. Cell Adhesion Assay

Two days before adhesion assay, WI26 cells were seeded in 24-well plates (500 μL/well) at a density of 3 × 10^5^ cells/mL and allowed to grow 24 h to confluence. The medium of THP-1 and WI26 cells was replaced by DMEM supplemented with 0.5% FBS and antibiotics 24 h before the adhesion experiment. The culture of THP-1 cells was centrifuged at 1000 rpm for 5 min. The supernatant was discarded. The THP-1 cells were re-suspended in DMEM with 10% FBS and antibiotics, then diluted to 5 × 10^5^ cells/mL. The medium of WI26 cells in 24-well plates was discarded to get a monolayer of WI26 cells in each well, then 500 μL THP-1 suspension was added into each well as previously published [38,39]. For the biotest groups, 30 μg/mL compound **1** or 50 μg/mL crude extract of *S. xiamenensis* culture was added in each well, respectively. For the control groups, 0.1% DMSO was used as solvent control for **1** and 0.01% DMSO as solvent control of crude extract, respectively, according to the different final DMSO concentration of **1** and crude extract. Two kinds of cells were co-cultured for 3 h, then the medium and the non-adherent THP-1 cells were removed by washing with PBS three times. Adherent THP-1 cells were quantified microscopically at 400-fold magnification in 4 random visual fields of each well and photographed with a digital camera (LY-WN-HPCCD, China) mounted on a microscope (Olympus, CKX31, Japan). Data are shown as means ± standard deviation (SD) of three independent experiments, each performed in triplicate.

### 3.9. Fibroblast Contraction Assay

WI26 cells at 70%–80% confluent were trysinized by 0.25% trypsin-0.02% EDTA solution and seeded at a density of 4 × 10^5^ cells/mL into 32 mm bacteriological plates (2 mL/dish) in DMEM supplemented with 10% FBS, antibiotics, sodium ascorbate (50 μg/mL), and containing 0.3 mg/mL of acid-extracted collagen I from newborn calf skin (IBFB, Leipzig, Germany) as previously described [40]. For the biotest groups, 30 μg/mL pure extract of compound **1** was added in each well; for the control groups, 0.1% DMSO was used as solvent control. The cultures were placed at 37 °C for 60 min to allow collagen polymerization, and then the gels from plates were released by tilting the plates slightly. Gradual lattice contraction was monitored by measuring the gel area of triplicate setups at successive time points up to 6 h. Data are shown as means ± standard deviation (SD) of three independent experiments, each performed in triplicate.

### 3.10. Statistical Analysis

Statistic differences were calculated using student’s paired *t*-test at a significance level of *p* < 0.05 to 0.001. 

### 3.11. Computational Methods

The QM calculations were carried out with the M06-2X hybrid meta DFT functional with the 6 − 31 + G(d,p) basis set using Gaussian 09. Conformations were fully optimized and characterized by harmonic vibrational frequency analysis. The solvation energies in DMSO were calculated with Truhlar’s SMD solvation model [41,42,43]. 

## 4. Conclusions

The investigation of anti-fibrosis small molecules derived from *S. xiamenensis* may represent an emerging pharmacological and therapeutic area for fibrotic diseases. The structure and absolute configurations of *N*-[[3,4-dihydro-3*S*-hydroxy-2*S*-methyl-2-(4′*R*-methyl-3′*S*-pentenyl)-*2H*-1-benzopyran-6-yl]carbonyl]-threonine (**1**) was fully elucidated by extensive spectroscopic data analyses, Mosher’s method, Marfey’s reagent and QM calculations. The multiple biological effects of **1** on lung fibroblast behavior were investigated, including proliferation, anti-contractile capacity and anti-adhesion with monocytes. These three bioactive aspects are involved in the construction of positive feedback circuits of excessive fibrosis. Developing leading compounds from marine natural products, aiming at these circuits may have targeted effects on fibrotic diseases.

## Supplementary Files

Supplementary File 1: PDF-Document (PDF, 535 KB)
