# Supplementary material for: Identification and Characterization of an Anti-Fibrotic Benzopyran Compound Isolated from Mangrove-Derived Streptomyces xiamenensis
Source: Mar Drugs. 2012 Mar 15;10(3):639–54. doi: 10.3390/md10030639 (PMC3347021; doi:10.3390/md10030639)
Supplement: Supplementary File 1: — PDF-Document (PDF, 535 KB) [file marinedrugs-10-00639-s001.pdf]

Supplementary Materials

Identification and characterization of an anti-fibrotic benzopyran compound isolated from mangrove-derived *Streptomyces xiamenensis*

Min-Juan Xu[1,†], Xiao-Jin Liu[2,3,†], Yi-Lei Zhao[4], Dong Liu[5], Zhen-Hao Xu[4], Xiao-Meng Lang[5], Ping Ao[1], Wen-Han Lin[5], Song-Lin Yang[2], Zhi-Gang Zhang[3*], Jun Xu[4*]

[1] Shanghai Center for Systems Biomedicine, Shanghai Jiao Tong University, Key Laboratory of Systems Biomedicine, Shanghai, 200240, P. R. China

[2] Department of Plastic Surgery, Shanghai Jiaotong University Affiliated Sixth People's Hospital, Shanghai, 200233, P. R. China

[3] State Key Laboratory of Oncogenes and Related Genes, Shanghai Cancer Institute, Ren Ji Hospital, Shanghai Jiao Tong University School of Medicine, Shanghai, 200240, P. R. China

[4] State Key Laboratory of Microbial Metabolism and School of Life Science & Biotechnology, Shanghai JiaoTong University, Shanghai, 200240, P. R. China

[5] State Key Laboratory of Natural and Biomimetic Drugs, Peking University, Beijing 100191, P. R. China

[†] These authors contributed equally to this work.

***** Author to whom correspondence should be addressed: E-Mail: xujunn@sjtu.edu.cn (J. X.); Tel.: +86-021-34207208 (J. X.); Fax: +86-021-34207205 (J. X.). E-Mail: zzhang@shsci.org (Z.-G. Zh.); Tel.: +86-021-34206763 (Z.-G. Zh.); Fax: +86-021-34206763 (Z.-G. Zh.).

Table of contents

	Page
● Figure S1. HPLC seperation of Marfey's derivatives of hydrolysed compound **1** and L/D-Thr.	3
● Table S1. HPLC seperation of Marfey's derivatives of hydrolysed compound **1** and L/D-Thr.	3
● Figure S2. ^{1}H NMR spectrum of compound **1** in DMSO-d_6.	4
● Figure S3. ^{13}C NMR spectrum of compound **1** in DMSO-d_6.	4
● Figure S4. HMQC spectrum of compound **1** in DMSO-d_6.	5
● Figure S5. HMBC spectrum of compound **1** in DMSO-d_6.	5
● Figure S6. COSY spectrum of compound **1** in DMSO-d_6.	6
● Figure S7. NOESY spectrum of compound **1** in DMSO-d_6.	6
● Figure S8. ^{1}H NMR spectrum of compound **1** in [D_4]methanol.	7
● Figure S9. IR spectrum of compound **1**.	7
● Figure S10. UV spectrum of compound **1**.	8
● Figure S11. CD spectrum of compound **1**.	8
● Figure S12. HRMS spectra of compound **1** (positive mode).	9
● Figure S13. HRMS spectra of compound **1** (negative mode).	10
● Data comparison of **1** and the know compound [14]	11

General

Resolution of 2,4-dinitrophenyl-5-L-alanine amide (DNPA) amino acids: DNPA-L-Thr, DNPA-D-Thr, and DNPA-hydrolysed **1** was resolved by HPLC on a Welch XB-C18 column (4.6 mm × 150 mm, 5 μm) with UV detection monitored at 340 nm. For mobile phase, 0.05% trifluoroacetic acid (TFA) and 1% methanol was used in both A and B solution. Mobile phase A and B consisted of 5% acetonitrile and 60% acetonitrile, respectively. Linear gradients started with 0% B and finished with 100% B in 45 min, plus 15 min equilibrium of system at the beginning.

Figure S1. HPLC seperation of Marfey's derivatives of hydrolysed compound **1** and L/D-Thr. Note: Retention time of FDAA: 41.3 min.

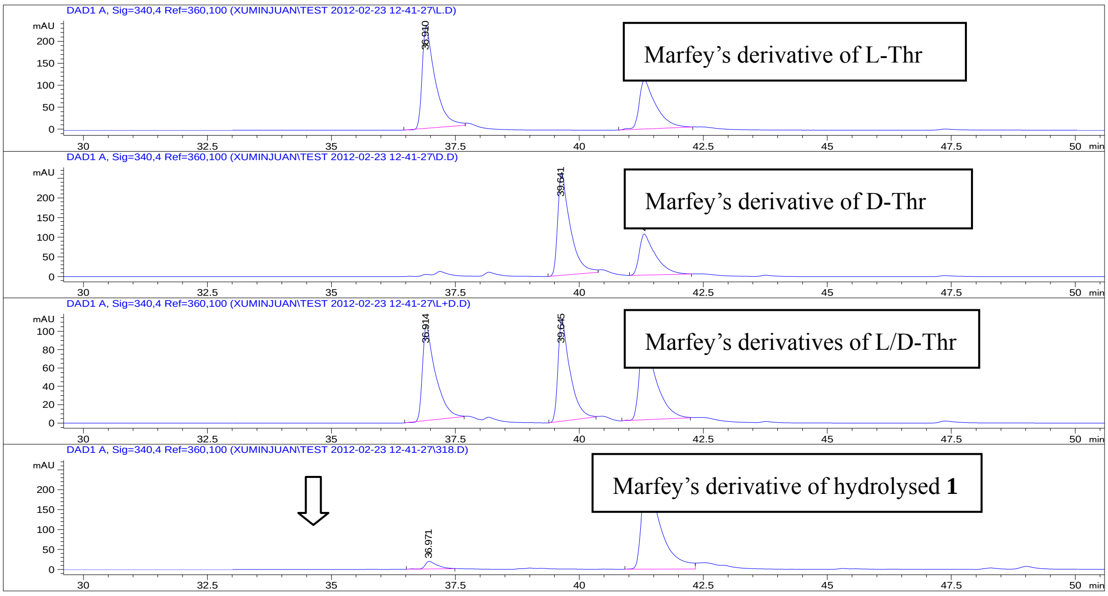

Table S1. HPLC seperation of Marfey's derivatives of hydrolysed compound **1** and L/D-Thr.

Sample	Retention time of Marfey's derivatives (min)	
	L	D
Thr	36.9	39.6
Hydrolysed compound **1**	36.9	--

Figure S2. ^{1}H NMR spectrum of compound **1** in DMSO-d_6.

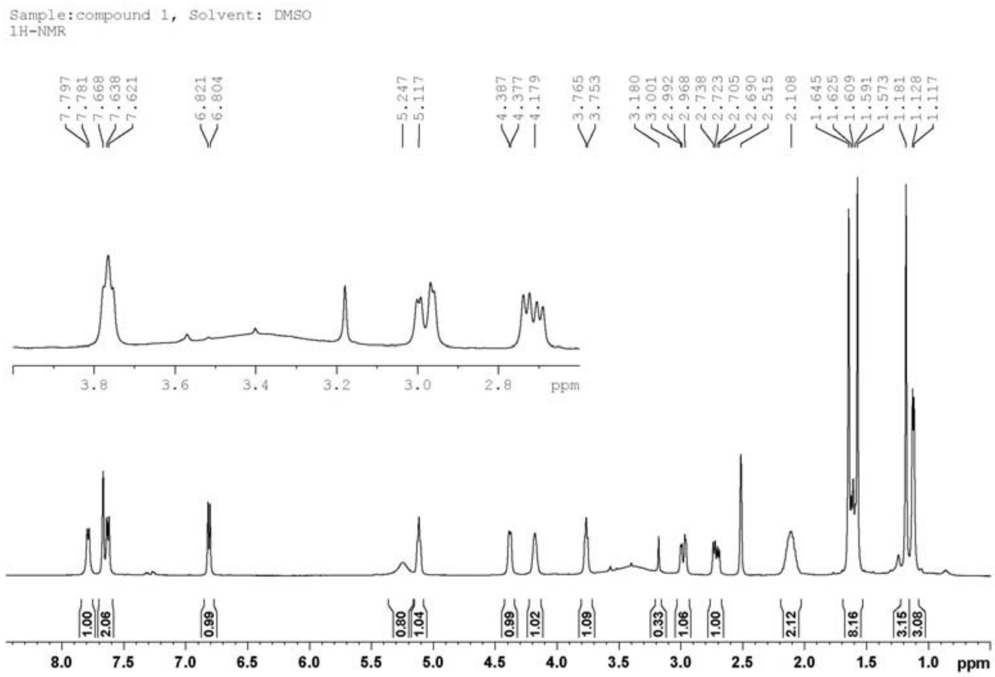

Figure S3. ^{13}C NMR spectrum of compound **1** in DMSO-d_6.

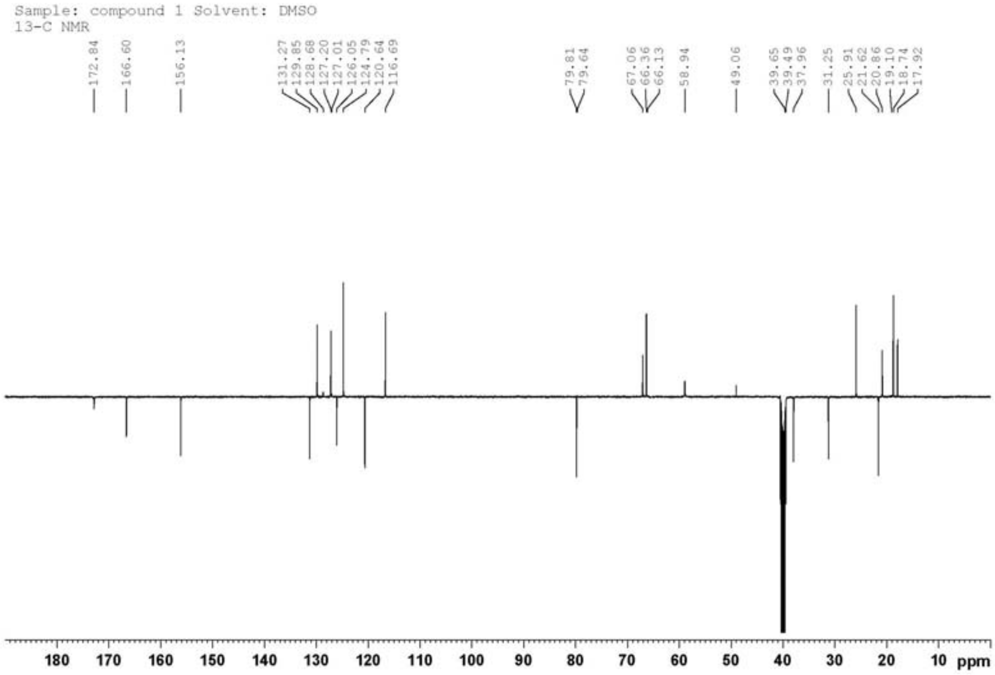

Figure S4. HMQC spectrum of compound **1**in DMSO-d_6.

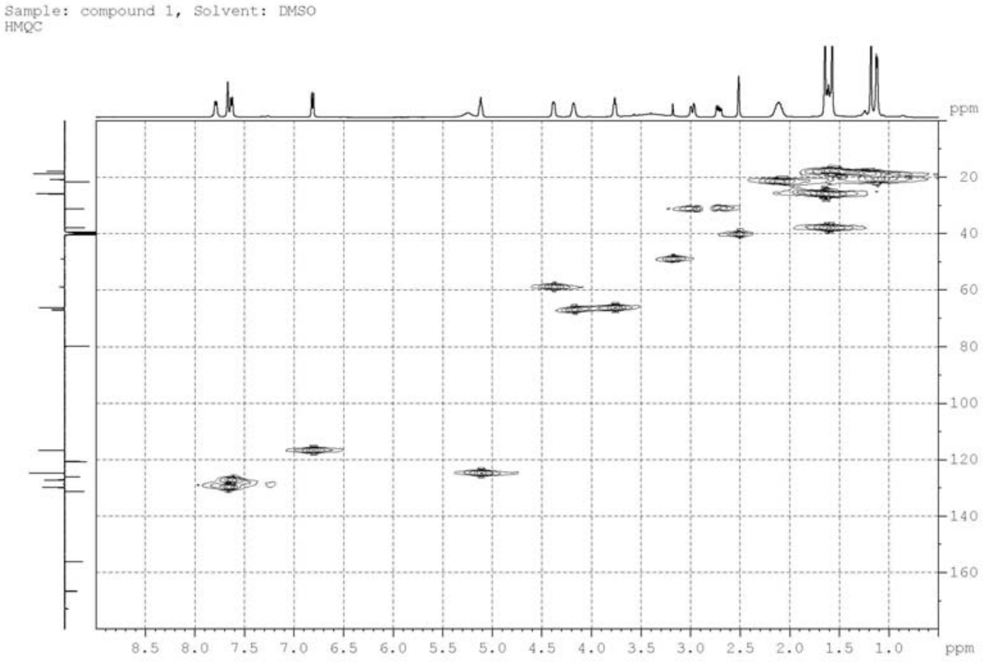

Figure S5. HMBC spectrum of compound **1** in DMSO-d_6.

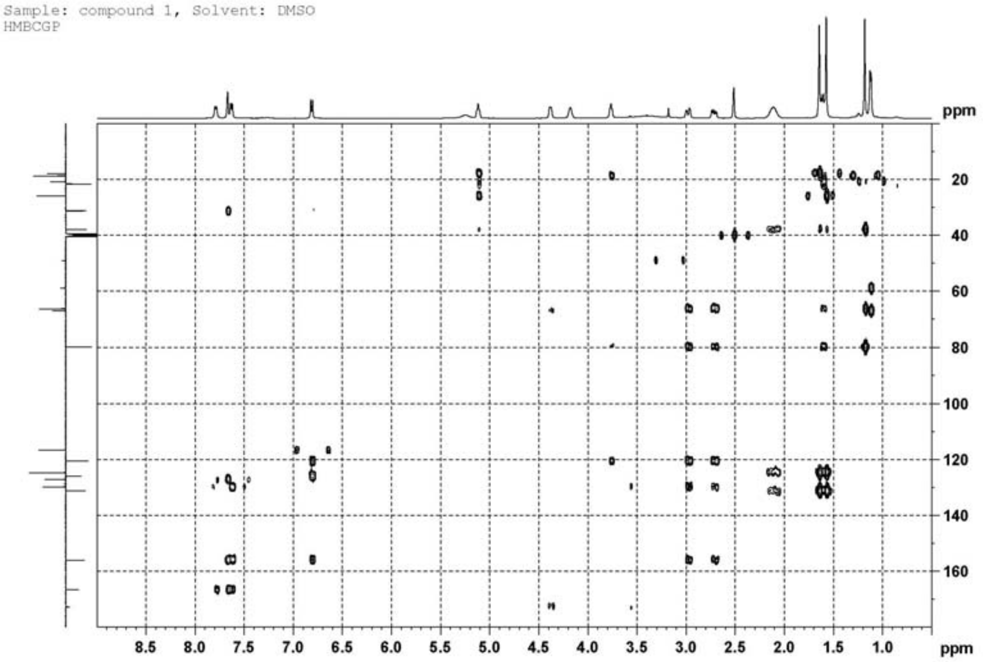

Figure S6. COSY spectrum of compound **1** in DMSO-d_6.

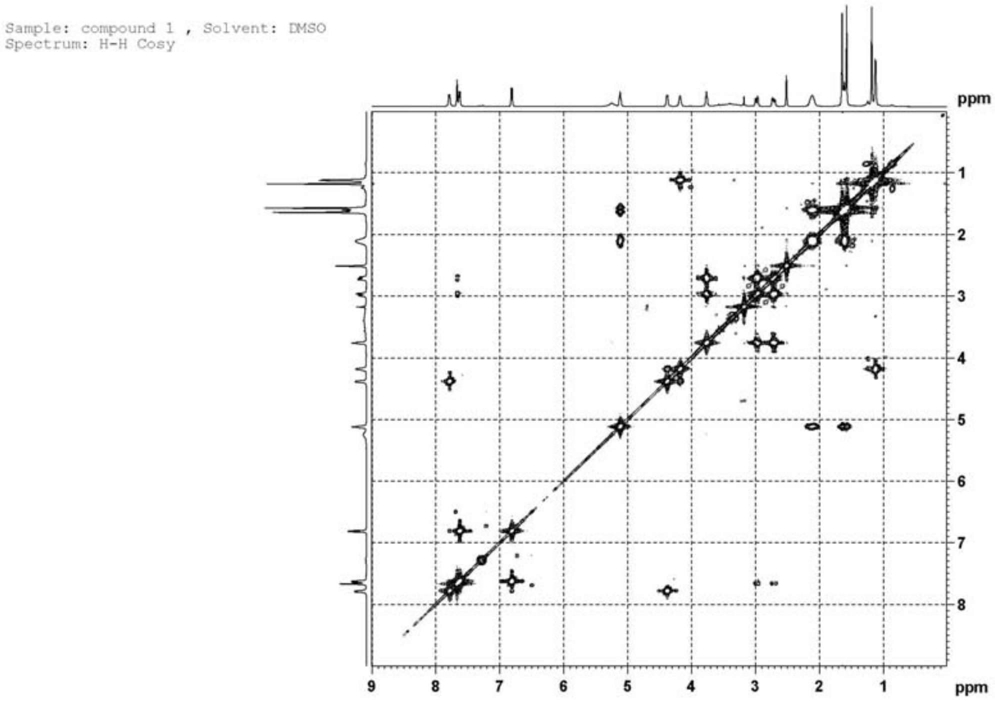

Figure S7. NOESY spectrum of compound **1** in DMSO-d_6.

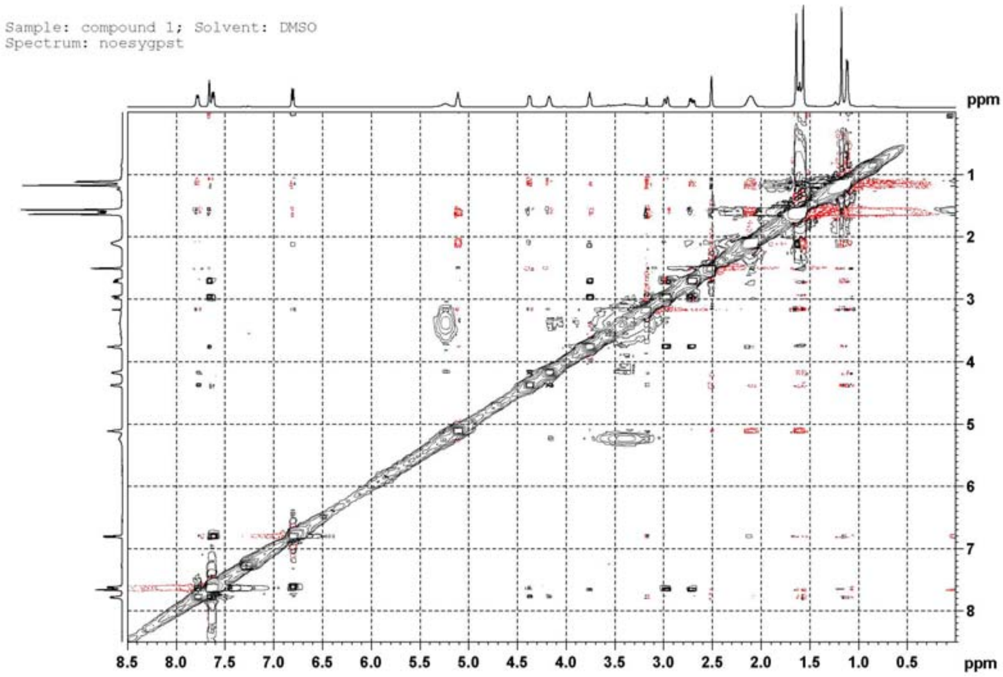

Figure S8. ^{1}H NMR spectrum of compound **1** in [D$_4$]methanol.

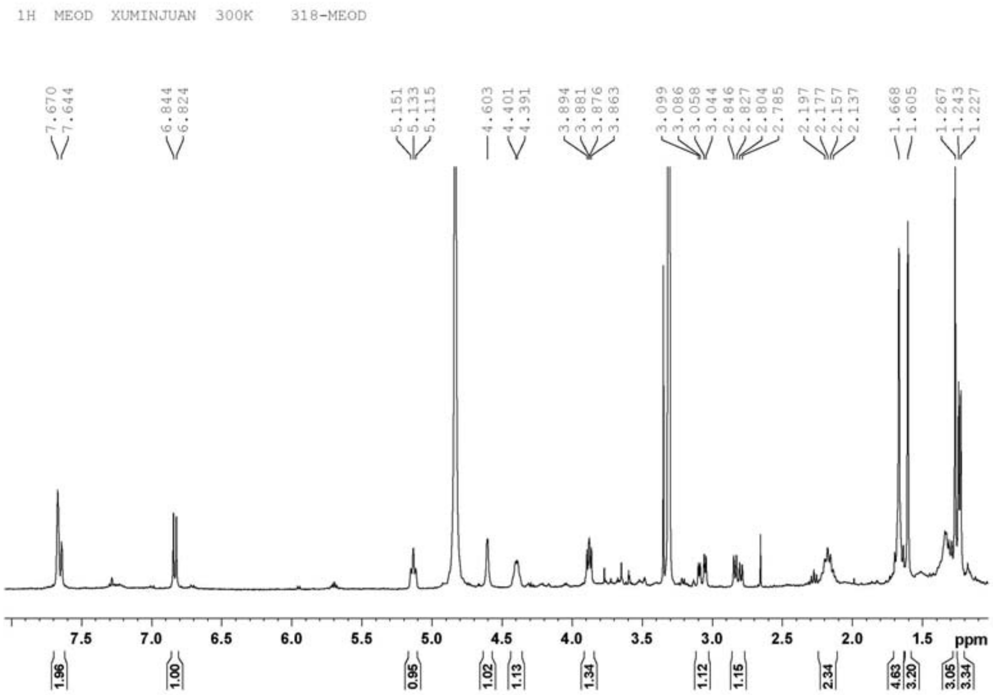

Figure S9. IR spectrum of compound **1**.

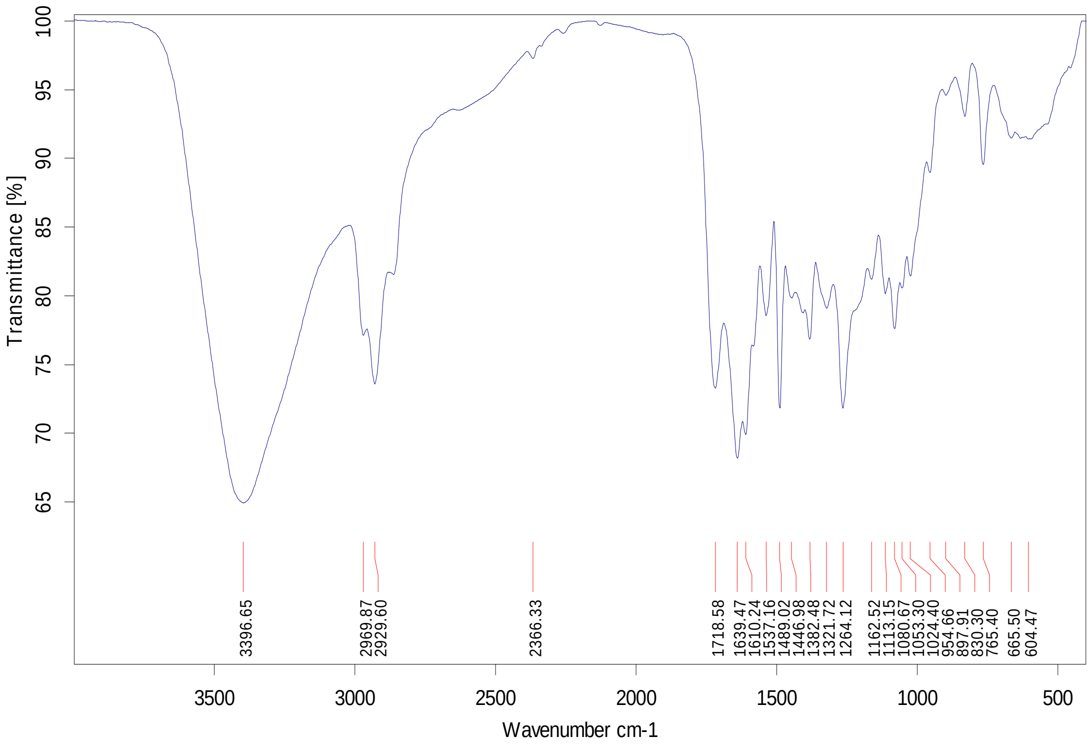

Figure S10. UV spectrum of compound **1** (online detection by HPLC).

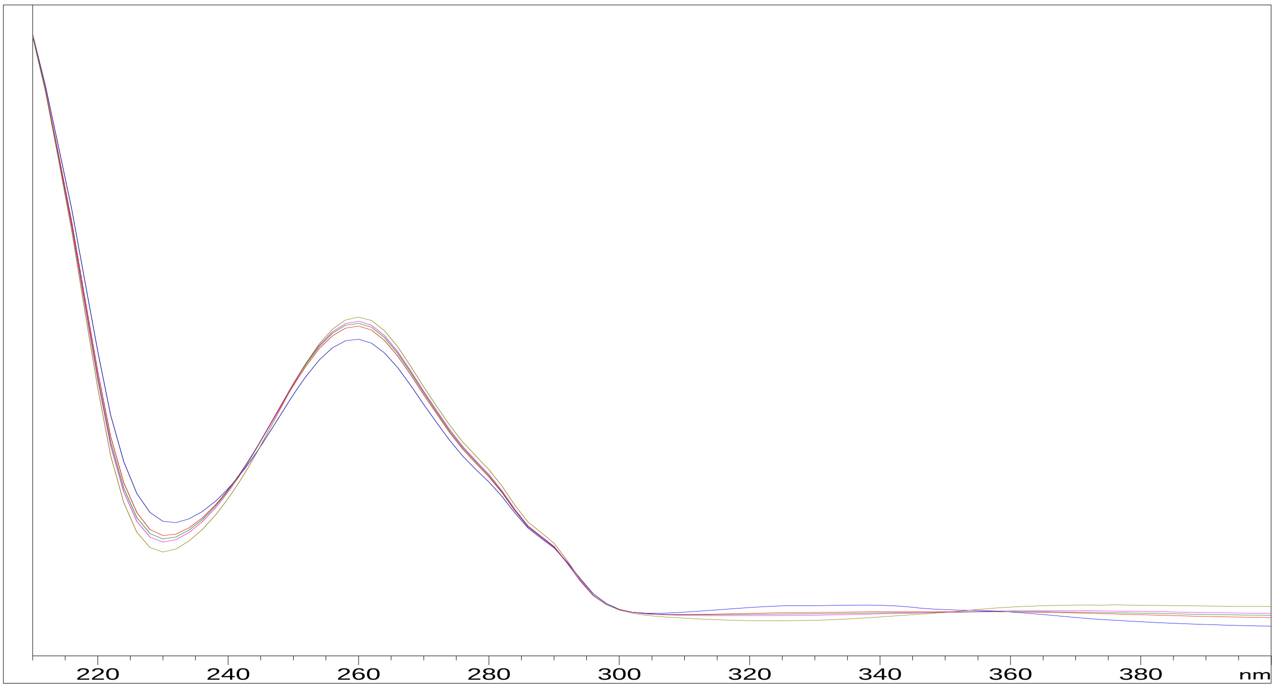

Figure S11. CD spectrum of compound **1**.

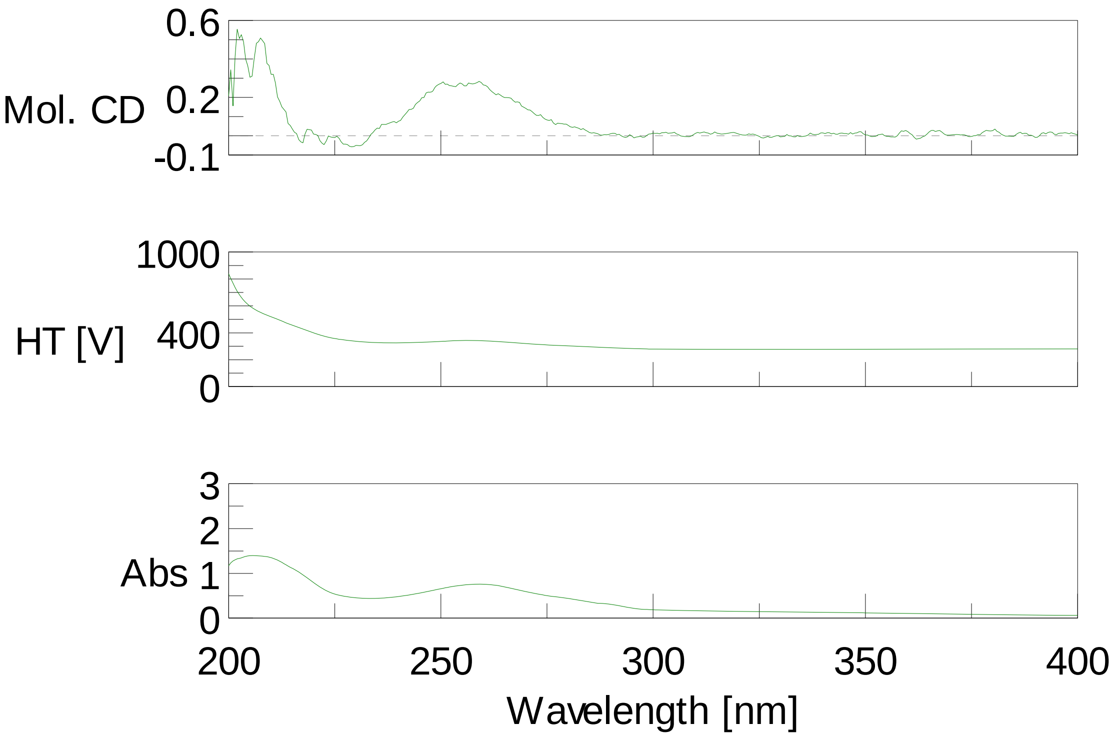

Figure S12. HRESIMS spectra of compound **1** (Positive mode).

Mass	Calc. Mass	mDa	PPM	DBE	i-FIT	Norm	Conf(%)	Formula
392.2069	392.2073	-0.4	-1.0	7.5	27.0	1.147	31.76	C21 H30 N O6
	392.2226	-15.7	-40.0	11.5	28.0	2.058	12.77	C25 H30 N O3
	392.1862	20.7	52.8	12.5	27.3	1.442	23.64	C24 H26 N O4
	392.2437	-36.8	-93.8	6.5	27.0	1.145	31.83	C22 H34 N O5

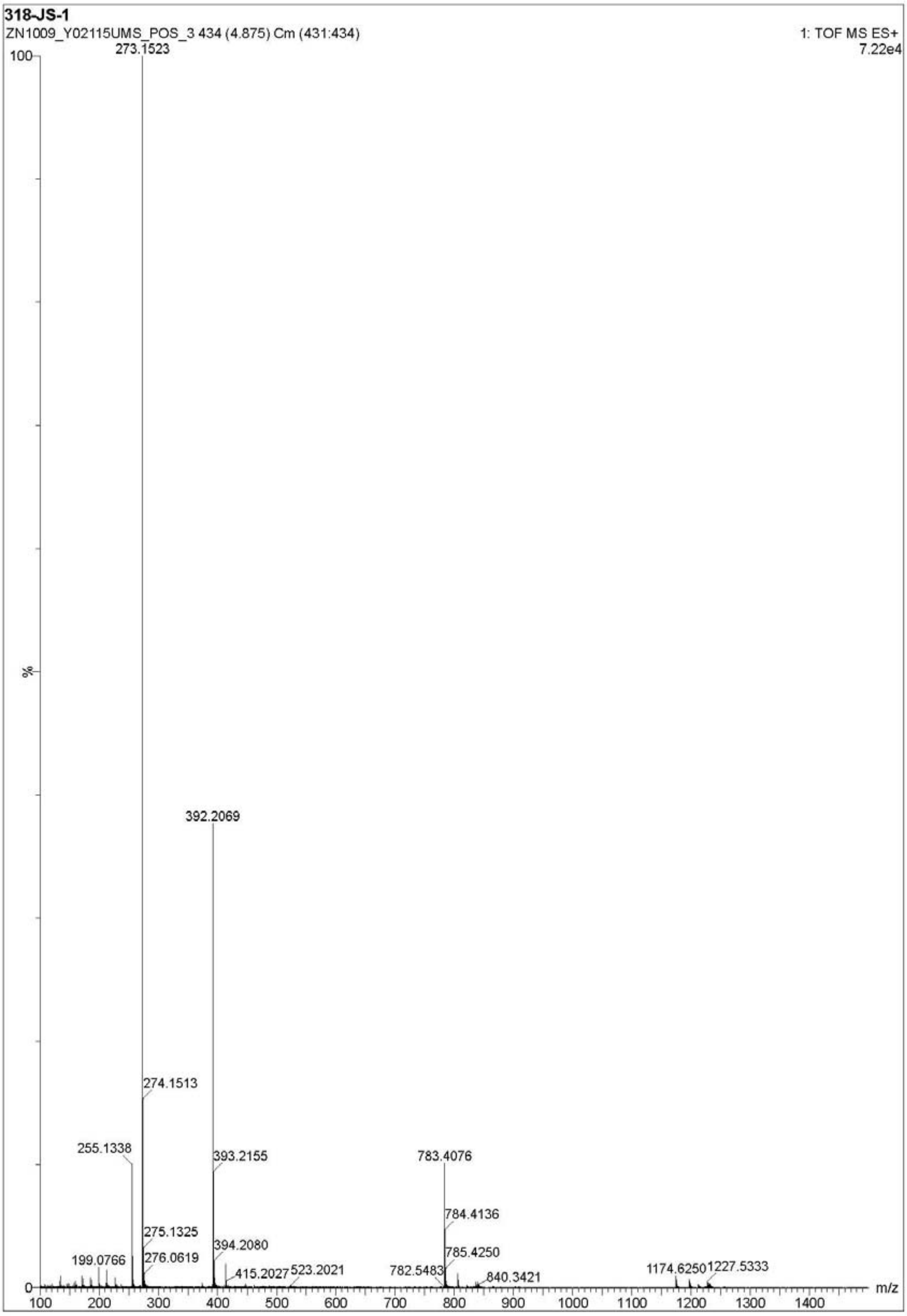

Figure S13. HRESIMS spectra of compound **1** (Negative mode).

Mass	Calc. Mass	mDa	PPM	DBE	i-FIT	Norm	Conf(%)	Formula
390.1886	390.1917	-3.1	-7.9	8.5	37.5	0.373	68.86	C21 H28 N O6
	390.1858	2.8	7.2	17.5	38.3	1.167	31.14	C28 H24 N O

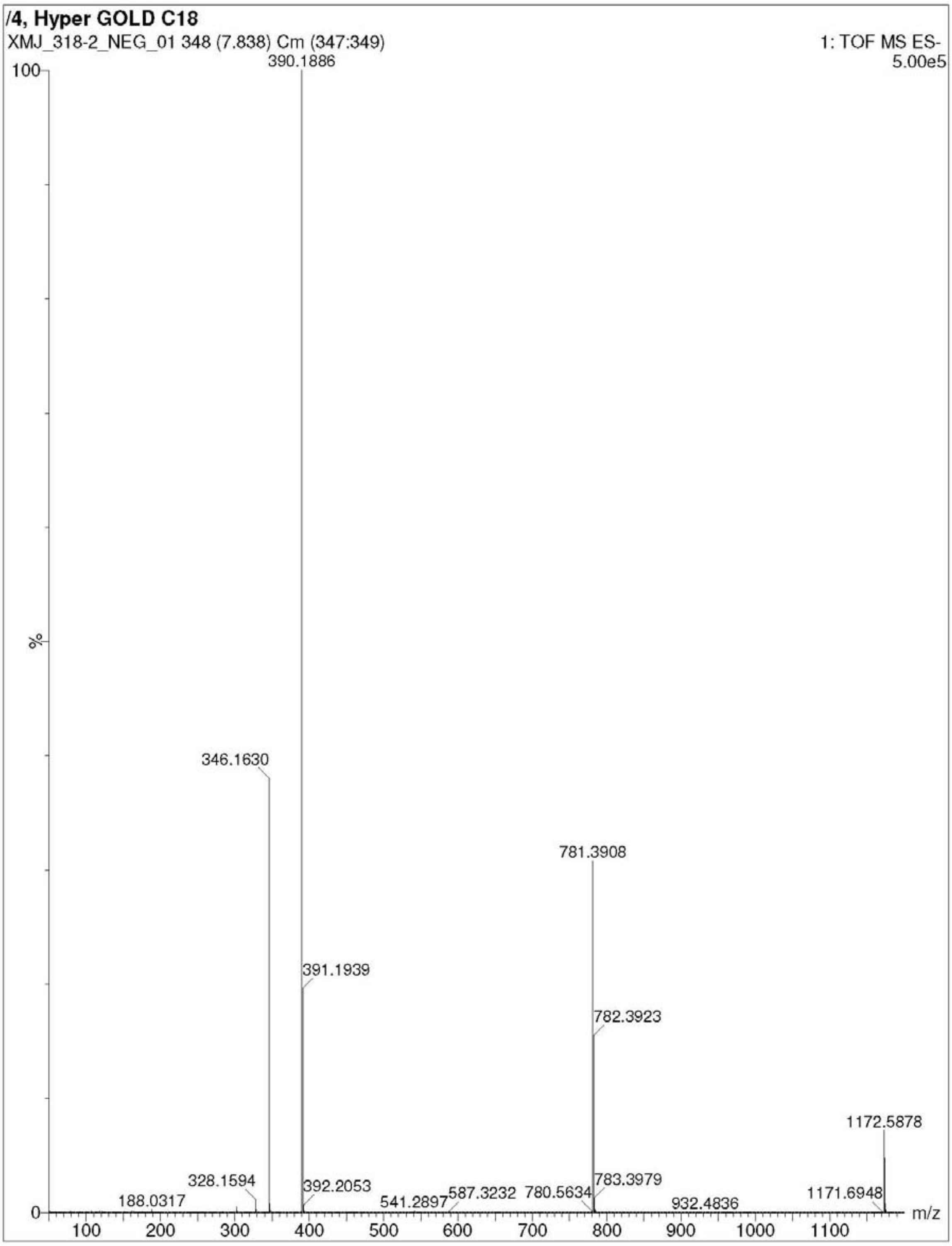

Data comparison of **1** and the know compound [14]

1) IR data from reference 14 (KBr): 3866, 3383, 2976, 2932, 1730, 1640, 1611, 1578, 1539, 1491, 1422, 1379, 1321, 1265, 1200, 1157, 1113, 1082, 1016, 952, 897, 833, 767, 667 cm-1.

IR data of compound **1** (KBr): 3396, 2969, 2929, 1718, 1639, 1610, 1579, 1537, 1489, 1446, 1382, 1321, 1264, 1201, 1162, 1113, 1080, 1024, 954, 897, 830, 765, 665 cm^{-1}.

2) ^{13}C-NMR data from reference 14 (CD_3OD): 178.3 s, 169.7 s, 157.9 s, 132.5 s, 130.8 d, 128.1 d, 126.8 d, 125.4 d, 121.4 s, 118.0 d, 80.5 s, 69.3 d, 68.3 d, 60.8 d, 38.9 t, 32.0 t, 25.9 q, 22.6 t, 20.1 q, 18.9 q, 17.7 q.

^{13}C-NMR data of compound **1** (CD_3OD): 170.0 s, 157.9 s, 132.5 s, 130.7 d, 128.0 d, 126.9 d, 125.3 d, 121.5 s, 118.0 d, 80.7 s, 68.9 d, 68.3 d, 60.8 d, 38.9 t, 32.0 t, 25.8 q, 22.6 t, 20.6 q, 18.9 q, 17.6 q. (The carbon signal of C-6 can't be detected due to limited amount. Please find the full set of carbon signals of compound **1** in Table 1 or in Figure S3.)

3) ^{1}H-NMR data from reference 14 (CD_3OD):7.67 (1H, s), 7.66 (1H, d), 6.83 (1H, d), 5.13 (1H, t), 4.63 (1H, d), 4.41 (1H, dq), 3.88 (1H, dd), 3.07 (1H, dd), 2.81 (1H, dd), 2.16 (2H, m), 1.66 (3H, s), 1.66 (2H, m), 1.60 (3H, s), 1.26 (3H, s), 1.21 (3H, d).

^{1}H -NMR data of compound **1** (CD_3OD): 7.67 (1H, s), 7.65 (1H, d), 6.83 (1H, d), 5.13 (1H, t), 4.60 (1H, d), 4.40 (1H, dq), 3.88 (1H, dd), 3.07 (1H, dd), 2.81 (1H, dd), 2.18 (2H, m), 1.67 (3H, s), 1.66 (2H, m), 1.60 (3H, s), 1.27 (3H, s), 1.23 (3H, d).

Reference 14: Kawamura, N.; Tsuji, E.; Watanabe, Y.; Tsuchihashi, K.; Takako, T. Benzopyran derivatives, their manufacture with *Streptomyces* species, and their use for treatment of asthma and rheumatoid arthritis. Daiichi Seiyaku Co., Ltd.; Mercian Corp.: Kyoto, Japan, 7 March 2000. Avaliable online:
http://worldwide.espacenet.com/publicationDetails/biblio?DB=EPODOC&II=0&ND=3&adjacent=true&locale=en_EP&FT=D&date=20000307&CC=JP&NR=2000072766A&KC=A
